# CD40 Cross-Linking Induces Migration of Renal Tumor Cell through Nuclear Factor of Activated T Cells (NFAT) Activation

**DOI:** 10.3390/ijms22168871

**Published:** 2021-08-18

**Authors:** Paola Pontrelli, Margherita Gigante, Federica Spadaccino, Giuseppe Stefano Netti, Marilisa Saldarelli, Luigi Balducci, Maddalena Gigante, Michele Battaglia, Walter J. Storkus, Giuseppe Castellano, Giovanni Stallone, Loreto Gesualdo, Elena Ranieri

**Affiliations:** 1 Department of Emergency and Organ Transplantation, Divisions of Nephrology and Urology, University of Bari, Piazza G. Cesare 11, 70124 Bari, Italy; paola.pontrelli@uniba.it (P.P.); michele.battaglia@uniba.it (M.B.); loreto.gesualdo@uniba.it (L.G.); 2 Department of Medical and Surgical Sciences, Divisions of Clinical Pathology and Nephrology, University of Foggia, Policlinico Riuniti, Viale L. Pinto, 71100 Foggia, Italy; gigantem@libero.it (M.G.); federica.spadaccino@unifg.it (F.S.); giuseppestefano.netti@unifg.it (G.S.N.); marilisa.saldarelli@unifg.it (M.S.); luigi.balducci@unifg.it (L.B.); mgigante72@gmail.com (M.G.); giuseppe.castellano@unifg.it (G.C.); giovanni.stallone@unifg.it (G.S.); 3 Department of Immunology, University of Pittsburgh School of Medicine, Pittsburgh, PA 15261, USA; storkuswj@upmc.edu

**Keywords:** renal cell carcinoma, CD40/CD40L crosslinking, cell migration, NFATs, integrin β1

## Abstract

CD40 crosslinking plays an important role in regulating cell migration, adhesion and proliferation in renal cell carcinoma (RCC). CD40/CD40L interaction on RCC cells activates different intracellular pathways but the molecular mechanisms leading to cell scattering are not yet clearly defined. Aim of our study was to investigate the main intracellular pathways activated by CD40 ligation and their specific involvement in RCC cell migration. CD40 ligation increased the phosphorylation of extracellular signal-regulated kinase (ERK), c-Jun NH (2)-terminal kinase (JNK) and p38 MAPK. Furthermore, CD40 crosslinking activated different transcriptional factors on RCC cell lines: AP-1, NFkB and some members of the Nuclear Factor of Activated T cells (NFAT) family. Interestingly, the specific inhibition of NFAT factors by cyclosporine A, completely blocked RCC cell motility induced by CD40 ligation. In tumor tissue, we observed a higher expression of NFAT factors and in particular an increased activation and nuclear migration of NFATc4 on RCC tumor tissues belonging to patients that developed metastases when compared to those who did not. Moreover, CD40-CD40L interaction induced a cytoskeleton reorganization and increased the expression of integrin β1 on RCC cell lines, and this effect was reversed by cyclosporine A and NFAT inhibition. These data suggest that CD40 ligation induces the activation of different intracellular signaling pathways, in particular the NFATs factors, that could represent a potential therapeutic target in the setting of patients with metastatic RCC.

## 1. Introduction

Renal cell carcinoma (RCC) is the most common tumor in the kidney. Although metastasized RCC has generally a bad prognosis, this tumor type may be sensitive to immune attack, with spontaneous regression of metastatic lesions reported in some cases [1,2].

CD40 is a type I membrane glycoprotein that belongs to the tumor necrosis factor receptor superfamily and represent an important T cell co-stimulatory molecule. The isolation of its ligand (CD40L/CD154), a type II transmembrane protein predominantly expressed in activated T cells, paved the way for the demonstration of a key role for CD40-CD40L interaction in the regulation of innate and adaptive immunity but also in the induction of tolerance.

CD40 is largely expressed in various types of carcinomas including breast, ovarian, nasopharyngeal, colon, lung, bladder and also RCC [3]. The widespread expression of CD40 in carcinomas indicates a possible role for this receptor in the pathogenesis of cancer [4]. In particular, the cross-linking of CD40 by CD40 ligand may promote the progression of different tumor types through the induction of cell motility and migration [5,6]. It has been demonstrated that in primary RCC cells [7], CD40 ligation drove proliferation and increased motility [3], although the intracellular mechanisms leading to this effect have not been yet well defined.

Structurally, CD40 comprises a 277 aminoacid protein with a large 193 aminoacid extracellular domain, a 22 aminoacid transmembrane region and a short 62 aminoacid cytoplasmic C-terminus. The cytoplasmic tale of CD40 lacks intrinsic kinase activity and signals through the recruitment of adaptor proteins of the TNF receptor-associated factor family (TRAF) and subsequent activation of different signaling pathways [8,9].

Different intracellular pathways activated by CD40 ligation can be responsible of cell motility [10]. In RCC lines the platelet-activating factor may play a role in cell migration [3] while in human multiple myeloma cells, the cross-linking of CD40 induces cell scattering through the phosphoinositol 3-kinase (PI3K)/Akt/NFkB pathway [5]. Various evidences suggest that cell motility can also depend upon integrins engagement [11,12] and subsequent activation of the intracellular signaling starting from integrin-linked kinase (ILK) following CD40 ligation [13]. Integrins are receptors for extracellular matrix (ECM) compounds like collagen or fibronectin structurally organized into heterodimeric transmembrane complexes of an α and a β subunit. The binding of integrins to their ligands induces association with the actin cytoskeleton [12]. Dependent on the state of cytoskeletal organization, this can lead to clustering of integrins into focal complexes including focal adhesion kinase (FAK). It has been largely demonstrated that integrins of the β1 family influence migration of cancer cells and the cell surface expression of β1 integrins is positively correlated with increased metastatic potential and invasiveness of renal tumor cells [14,15]. In particular, β1 integrins and FAK play a critical role in the regulation of migration of renal cancer cells through the activation of protein kinase C [16].

In the analysis of the complex array of signaling pathways that regulate cell migration and cancer invasion, specific role for NFAT (nuclear factor of activated T cells) transcription factors has been demonstrated [17]. NFAT is a family of five transcription factors, four of which are calcineurin dependent (NFATc1, NFATc2, NFATc3, NFATc4) while NFAT5 is controlled by osmotic stress, all essential for T cells activation. In breast cancer cells, inhibition of NFAT pathway, blocks cell motility and invasion [18]. Jauliac et al. reported that NFATs are targets of α_6_β_4_ integrin signaling and promote carcinoma invasion [19].

In the present study we investigated the different intracellular signaling pathways activated by CD40 cross-linking and their possible involvement in cell motility. We observed that NFATs are relevant factors involved in RCC cell migration and could represent a potential target for therapeutic approaches.

## 2. Results

### 2.1. Effect of CD40 Ligation on Apoptosis and Cell Proliferation in RCC Cell Lines

**Figure 1:**
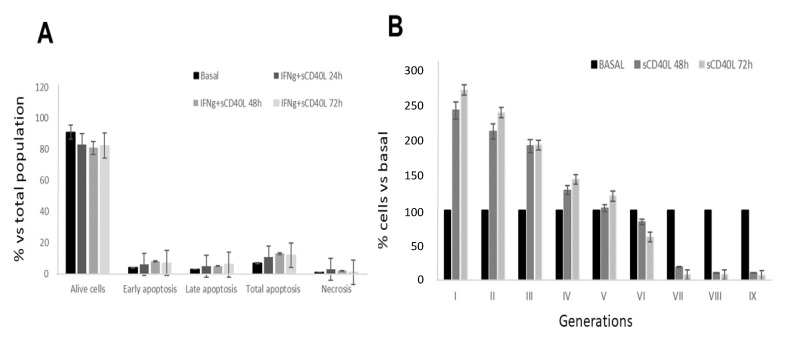
**Effect of CD40 ligation on cell apoptosis** (**A**) **and cell proliferation** (**B**) **in RCC cell lines.** (**A**) RCC cells were preincubated for 24 h with 500 UI/mL IFN-γ and then stimulated with sCD40L and sCD40L enhancer for 24, 48 and 72 h. Cells were analyzed by flow cytometry after staining with Annexin V-FITC and 7-AAD. Results were expressed as percentage of each distinct population (alive cells, apoptotic cells -early, late and total- and necrotic cells) versus the total cell number. The figure is representative of three experiments on three different RCC lines and there is no statistically significant difference over the conditions. (**B**) RCC cells were pre-incubated with 5 μM CFSE for 48 h, then pre-incubated for 24 h with 500 UI/mL IFNγ and stimulated with sCD40L and sCD40L enhancer. CFSE was detected in FL1 with a logarithmic amplifier. The figure is representative of three experiments on three different RCC lines.

It was previously reported that CD40 ligation stimulated cell proliferation and motility in RCC [3]. However, the specific molecular mechanisms induced by CD40 ligation on RCC cells have not been completely investigated. In order to explore the molecular mechanisms and intracellular pathways underlying these processes, we analyzed cell proliferation on nine RCC cell lines. First of all, we evaluated the expression of CD40 on these RCC cells lines within the first two passages of culture. As reported in Table 1, the RCC cell lines showed a large variability of CD40 surface expression as evaluated by flow cytometry (mean%: 42.97 ± 39.43). Thus, we focused on cell lines expressing CD40 at levels higher than 43%. The pre-incubation of the selected RCC cell lines with sCD40L and the enhancer for 24, 48 and 72 h respectively, had no significant effect on cell apoptosis as showed in Figure 1A. On the other hand, CD40-CD40L cross-linking promoted cell proliferation of RCC cells, as demonstrated with CFDA-SE assay by the decrease of CFSE content over time compared to basal condition in RCC cell lines stimulated with sCD40L at different time points (Figure 1B).

### 2.2. CD40 Ligation Activates Different Signalling Pathways in RCC Cell Lines

**Figure 2:**
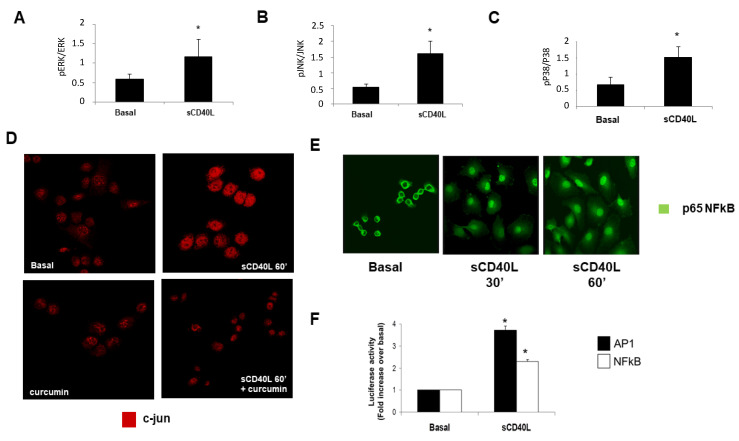
The effect of CD40 ligation on the activation of MAPK (ERK, JNK, P38) and of nuclear transcription factors (AP-1 and NFkB) in RCC cell lines. (**A**–**C**) Quiescent RCC cell lines were stimulated with sCD40L (100 ng/mL) and sCD40L enhancer (1 μg/mL) for 60 min and then lysed in RIPA buffer. Equal amounts of protein from each cell lysate (40 μg) were separated by SDS PAGE, transferred onto nitrocellulose membrane, and then analyzed by western blotting for the expression of p-ERK1/2, p-JNK or p-P38 normalized to ERK1/2, JNK1 or P38 respectively. The figure is representative of three experiments. * *p* < 0.005 vs. basal. (**D**) Jun activation in RCC cell lines in the presence or absence of curcumin, a specific JNK-AP1 inhibitor, was analyzed by confocal microscopy. The figure is representative of three experiments on different RCC cell lines. Cells magnification 60X. (**E**) NFκB activation was analyzed by confocal microscopy using an antibody recognizing the p65 subunit of the nuclear factor. RCC cells were stimulated with sCD40L (100 ng/mL) and sCD40L enhancer (1 μg/mL) or sCD40L enhancer alone in the basal condition, for 30 and 60 min respectively and then processed as described in methods. The figure is representative of three experiments. Cells magnification 60×. (**F**) Confluent, quiescent RCC cells were trypsinized and 5 × 10^6^ cells were resuspended in half milliliter medium containing 7.5 μg of pLucNF-kB vector and 2.5 μg of pCMVβGal. Electroporation was carried out as described in methods. In separate sets of experiments quiescent 5 × 10^6^ RCC cells were resuspended in half milliliter medium and 0.4 μg of pLuc3XAP1 were added. Luciferase activity was measured as described in methods. The figure is representative of three experiments. * *p* < 0.01 vs. basal.

To define the intracellular signaling pathways potentially activated by CD40 cross-linking and involved in cell motility, we focused our attention on the pathways involving the family of the mitogen-activated protein kinase (MAPK: JNK, ERK, p38), the nuclear factor AP-1 and the activation of NFκB. We observed that CD40 crosslinking on RCC cells induced an increase in different members of the MAPK family, as was described also in normal renal tubular cells [20]. CD40 stimulation in RCC cell lines, increased the phosphorylation with subsequent activation of extracellular signal-regulated kinase (ERK) (Figure 2A), c-Jun NH(2)-terminal kinase (JNK) (Figure 2B) and of p38 MAPK (Figure 2C). Moreover, CD40 crosslinking and subsequent JNK activation, induced also the nuclear migration of c-jun, a component of the activator protein 1 (AP-1), and this effect was inhibited by curcumin, a specific JNK-AP1 inhibitor (Figure 2D). We then investigated the effect of CD40L on the specific activation of the nuclear factors NFkB and AP-1. CD40 cross-linking caused a significant increase in NFκB nuclear translocation, as confirmed by confocal microscopy using a monoclonal antibody specifically recognizing the NFκB subunit p65. The incubation of RCC with soluble CD40L induced a significant and time-dependent nuclear translocation of p65, when compared to basal condition, already after 30 min of stimulation (Figure 2E). NFkB and AP1 activation were also confirmed by luciferase activity after transient transfection of RCC lines with an expression vector containing the luciferase cDNA under the control of three AP1 or NFκB consensus sequences (Figure 2F).

### 2.3. RCC Cell Proliferation and Motility Induced by CD40 Ligation Depends on NFAT Activation

**Figure 3:**
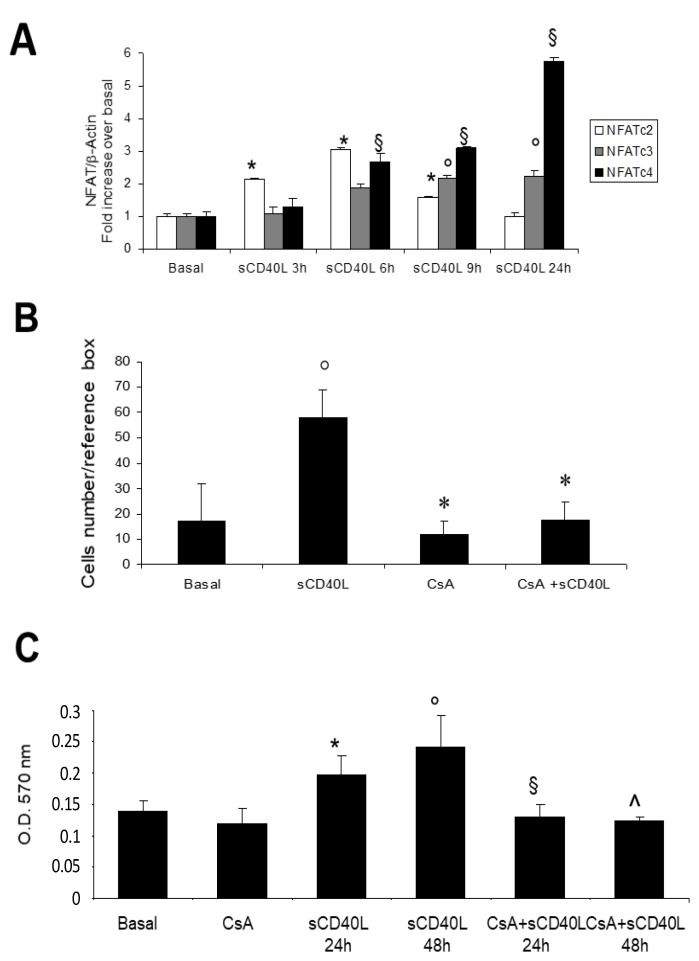
(**A**) **CD40L-induced NFATs expression in RCC cell lines:** Quiescent RCC cell lines were stimulated with sCD40L (100 ng/mL) and sCD40L enhancer (1 μg/mL) for the indicated time periods and then lysed in Trizol. RNA expression was investigated by real time PCR. The figure is representative of three experiments. *, ^§^, ° *p* < 0.001 vs. basal. (**B**) **The effect of NFATs inhibition by cyclosporine A on sCD40L-induced RCC cell migration.** The number of cells migrated into the denuded area in basal condition (sCD40L enhancer 1 μg/mL alone), after stimulation with sCD40L (100 ng/mL) and sCD40L enhancer (1 μg/mL) and after pre-incubation with cyclosporine A (CsA) and subsequent stimulation with sCD40L and enhancer for 24 h at 37 °C, was determined by counting, as described in methods. Results were expressed as the number of cells present in the reference box (mean ± SD of three experiments on different RCC lines), normalized to the level of total migration in the basal population. ° *p* = 0.03 vs. basal; * *p* = 0.01 vs. sCD40L. (**C**) **The effect of NFATs inhibition by cyclosporine A (CsA) on CD40L-induced cell proliferation in RCC cell lines.** MTT assay was performed as described in methods. The figure is representative of three different experiments on different RCC lines. * *p* < 0.01 vs. basal; ° *p* < 0.07 vs. basal; ^§^ *p* < 0.05 vs. sCD40L 24 h; ^ *p* < 0.05 vs. sCD40L 48 h.

It has been described that cell motility and invasion depend, at least in part, on the NFAT transcription factors [15,16]. Thus, we investigated the effect of CD40 crosslinking on NFAT gene expression in RCC. CD40 ligation on RCC lines induced a significant increase in mRNA abundance of NFATc2 with the highest peak after six hours of incubation, while NFATc3 and NFATc4, increased in a time dependent manner until 24 h (Figure 3A).

To further assess the role played by NFAT factors in RCC cell migration induced by sCD40L, we analyzed the specific effect of calcineurin/NFAT inhibitor, cyclosporin A, [21,22] on cell motility by a migration assay. Interestingly, we observed that cell motility, significantly induced by CD40 crosslinking on RCC cell lines, was completely abolished by pre-incubation with cyclosporin A (Figure 3B).

Moreover, CD40-CD40L cross-linking promoted cell proliferation of RCC cells, as demonstrated by MTT assay and CD40L-induced cell proliferation was significantly reduced by Cyclosporin A (Figure 3C), thus suggesting that NFAT factors play a significant role in this process.

**Figure 4:**
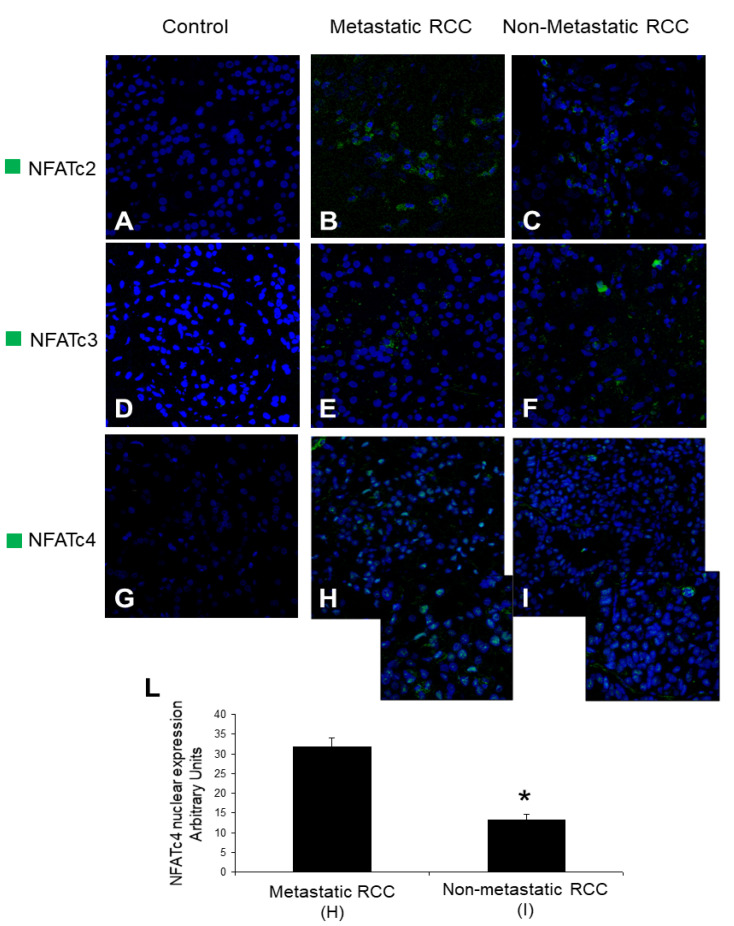
**NFATs expression on tumor tissues of RCC patients.** (**A**–**I**) All the images show the tissue at 40× magnification (big square) and 60× magnification (small square). Control: normal part of kidney tissue. The nuclei were stained with TO-PRO-3. (**L**) Quantification of NFATc4 nuclear expression. Results are expressed as mean ± SD. Three metastatic patients and three non-metastatic patients were evaluated. The nuclei were stained with TO-PRO-3. * *p* = 0.001 vs. metastatic patient.

The results obtained in vitro prompted us to investigate on the pathogenic role of NFAT in native material in RCC patients who developed metastases compared to those without metastases at least after 39 months from the diagnosis. NFATc2 (Figure 4B,C), NFATc3 (Figure 4E,F) and NFATc4 (Figure 4H,I) were all expressed at highest levels on biopsies of RCC tissues compared to normal tissues (Figure 4A,D,G respectively). Interestingly, for NFATc2 (Figure 4B,C) and NFATc3 (Figure 4E,F) no significant difference was observed among those patients who developed metastasis (Figure 4B,E respectively) compared to those who don’t (Figure 4C,F), at least after 39 months. Only in the case of NFATc4 we observed an increased expression at the nuclear level (Figure 4H,I), suggesting its functional activation. Moreover, we observed a significant higher expression of NFATc4 at nuclear level in those RCC patients who developed metastases (Figure 4H,L) compared to those patients without metastases (Figure 4I,L).

### 2.4. CD40 Ligation Promoted RCC Proliferation Acting on Cytoskeleton Organization

**Figure 5:**
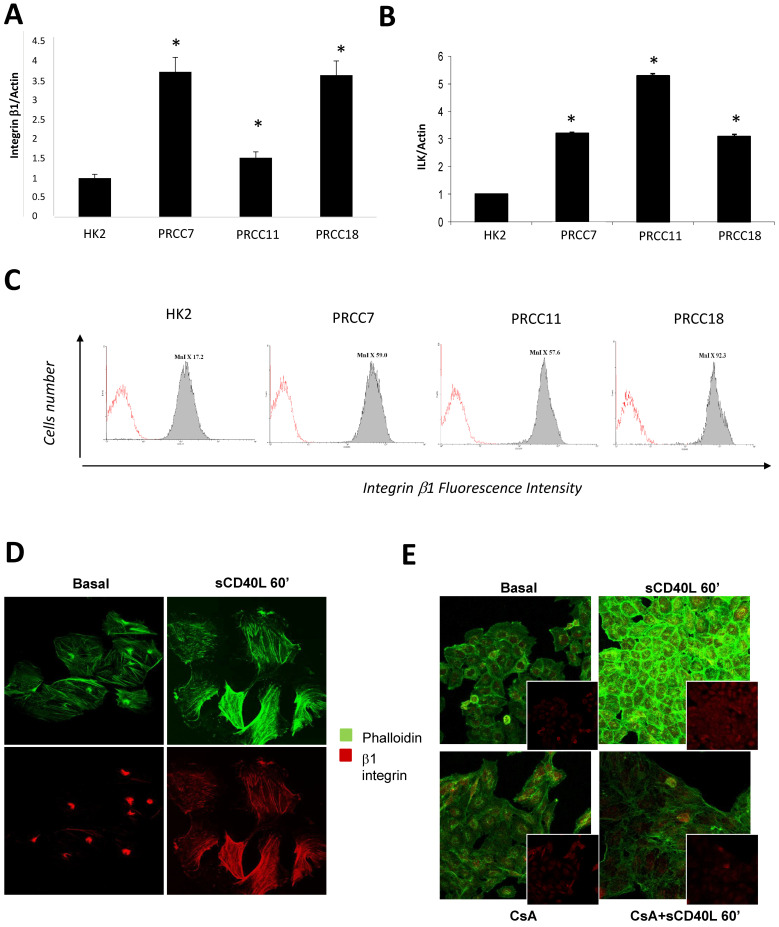
(**A**) **Effect of CD40 ligation and NFATs inhibition on β1 integrin and ILK expression and distribution.** (**A**,**B**) β1-integrin (**A**) and ILK (**B**) gene expression in RCC cell lines and HK2 cells. Real time PCR was performed as described in methods. * *p* < 0.05 vs. HK2 cells; (**C**) Flow cytometric expression of β1-integrin on HK2 cells and different RCC cell lines. MnI (Mean fluorescent intensity) for each condition is reported in each box. (**D**,**E**) β1-integrin and falloidin expression in basal conditions and after CD40 ligation as well as in the presence or absence of cyclosporine A, were evaluated by confocal microscopy as described in methods. Images magnification 60×. The figure is representative of three experiments.

To further evaluate the mechanisms activated by CD40 ligation on RCC cell lines and involved in cell proliferation, we moved to analyzing the cytoskeleton organization and integrins distribution. It has been demonstrated that β1 integrin plays an important role in the invasive phenotype of different tumor cell lines and also in RCC cells [14]. In our RCC cell lines we observed a significant increase in β1-integrin expression (Figure 5A) and ILK expression (Figure 5B) evaluated by real time PCR compared to tubular cells. By flow cytometry we also confirmed an increased expression of β1-integrin on the surface of RCC cell lines when compared to tubular cells (Figure 5C). Interestingly, CD40 crosslinking on RCC cells induced a specific change in actin fibers organization, as revealed by phalloidin staining after 60 min of incubation with sCD40L, and an increase in the expression and distribution of integrin β1 along all the cell surface (Figure 5D). This effect was inhibited by cyclosporin A, the NFATs inhibitor, that caused a reduction in CD40 ligation-induced integrin and actin expression and distribution, leading them to basal condition (Figure 5E).

## 3. Discussion

Co-stimulatory signaling via CD40/CD40L interaction may have several effects on dendritic cells, macrophages, B cells, epithelial and endothelial cells, and CD40-CD40L interaction plays a relevant role in the regulation of the immune response in different organs included the kidney [23]. The widespread expression of CD40 on different tumor types, including RCC, suggests that CD40 ligation can also influence tumor development [21]. The cross-linking of CD40 by CD40 ligand activates different signaling pathways in renal cell carcinoma cells [22] and in particular can influence proliferation and motility [3] thus playing a possible role also in the development of metastasis.

CD40-CD40L interaction transduces intracellular signals through TRAF (TNF receptor associated factors)-dependent and independent mechanisms and further downstream by different pathways and transcription factors. In melanoma cells, interference with nuclear factor kappa B and c-Jun NH2-terminal kinase signaling induced by TRAFs, inhibits myeloma cell proliferation [24]. In line with these results, in RCC cell lines, we also observed that CD40 ligation was able to induce an increased activation of the mitogen-activated protein kinase (MAPK) ERK, c-Jun N-terminal kinase (JNK) and p38 MAPK, together with the activation of key transcription factors, such as NFkB and AP-1, that represent key signaling pathways involved in proliferation and tumorigenesis [25].

Other than MAPK, CD40 ligation was also able to induce the activation of NFAT transcription factors. Kim et al. found an increased expression of NFATc1 in RCC tissues compared to normal tissues and shown that its inhibition reduces cell proliferation and tumor progression [8]. Küper et al. demonstrated that also NFATc5 plays an important role in RCC proliferation and migration [26]. Also, in prostate cancer the inhibition of NFAT by Cyclosporin A reduces cell viability and migration in tumor cell lines [27]. In our RCC cell lines, we demonstrated that CD40 engagement by CD40 ligand was able to significantly increase the transcription of different members of the NFAT family, in particular NFATc2, NFATc3 and NFATc4. Interestingly, CD40L-induced cell proliferation and migration of RCC cells were specifically inhibited by Cyclosporin A, which inhibits NFAT activity, thus underlying the role of NFAT factors in these processes induced by CD40 ligation.

Interestingly, by the analysis on RCC tissues, we observed an up-regulation of NFATs factors in tumor tissues when compared to normal tissue, however only NFATc4 was revealed at nuclear level, meaning its real activation in the tumor cells, and was up-regulated in metastatic RCC compared to non-metastatic tumor tissue. The limitations of the analyzed cohort are represented by non-metastasized samples that were all T1 stage and not high number of patients enrolled. Obviously further validations are needed to be performed in a large cohort of RCC patients. We did not evaluate CD40 expression in metastatic and non-metastatic tissues, however Weiss et al. also reported the up-regulation of CD40 in metastatic RCC [28]. Moreover, our in vitro results supported the relevant role played by CD40 ligation in the induction of NFAT factors.

It is known that RCC has a higher tendency to metastasize and it is well known that altered levels in integrin expression are closely involved in gain of tumorigenesis and metastatic ability in cancer cells. Breuksch et al. reported that integrin α5 was highly expressed in RCC tissues particularly in high-grade tumors highlighting its significant role in cancer progression. In addition, the analysis of the pathways revealed a correlation between integrin α5 expression and the increased activity of ERK [29]. In line with these evidences, we also observed an increased expression of Integrin β1 and of ILK kinase in our RCC cells compared to tubular cells. It has been demonstrated that ILK expression correlates with the severity of RCC and is also involved into the metastatic process [30,31]. We then investigated the effect of CD40 ligation on integrin β1 expression and distribution and the role of NFAT signaling into this process. Integrin receptors not only provide a link with actin cytoskeleton, but also serve as signaling receptors affecting cell behavior and several pathways of gene expression. Interestingly CD40 ligation induced a re-organization of actin fibers as well as of integrin β1 distribution in RCC cell lines. Of note, we observed that sCD40L-increased integrin β1 distribution and was affected by cyclosporin A, thus supporting the relevant role played by NFAT factors in integrins expression thus influencing RCC cell motility and invasiveness. Several in vitro experiments indicate that β1 integrin plays a critical role in the invasive phenotype of HCC cell lines [14] but also in renal carcinoma cells [15].

The knowledge of the different mechanisms activated by CD40 ligation in RCC patients will help us to identify novel approaches in the treatment of RCC. CD40 agonists represent today the novel frontier in the treatment of this carcinoma [32], since CD40 ligation plays an important role also in neo-vascularization of tumors [33] and anti-angiogenesis and immunotherapy represent next-generation approaches in the treatment of renal cell carcinoma [34]. Moreover, CD40 engagement can also enhance the immunostimulation of dendritic cells and effector cells against human RCC [24].

In conclusion, these data suggest that NFAT factors play a significant role in RCC cell scattering and migration through CD40 crosslinking and could represent a potential therapeutic target in the setting of patients with metastatic RCC.

## 4. Materials and Methods

### 4.1. Reagents

Fetal bovine serum (FBS), DMSO, hyaluronidase, collagenase IV, Dnase I and curcumin were obtained from Sigma Cell Culture (Milan, Italy). RPMI 1640 Medium, Penicillin/streptomycin, L-glutamine were from Life Technologies (Milan, Italy).

Recombinant human soluble sCD40L and sCD40L enhancer were from Alexis (Lausen, Switzerland) and were used at the same concentrations described in literature [3]. Cyclosporin A was from Novartis (Basel, Switzerland). All the experiments were performed in the presence of the crosslinking antibody (sCD40L enhancer) at basal condition.

The pLuc3XAP-1 vector (containing the firefly luciferase cDNA under the control of three AP-1 consensus sequence) [20] was kindly provided by Dr. C. Glass (University of Southern California at San Diego). The pLucNF-kB vector (containing the firefly luciferase cDNA under the control of three NF-kB consensus sequences) was kindly provided by Dr M. Fresno [35]. The dual luciferase assay kit was from Promega (Milan, Italy). The monoclonal anti-NFκB p65 antibody, the rabbit polyclonal anti-JNK1, the monoclonal anti p-JNK, the polyclonal anti-ERK1, the monoclonal anti-p-ERK, the polyclonal anti-P38, the monoclonal anti-p-P38, the monoclonal anti-c-jun were obtained from Santa Cruz Biotechnology Inc. (Santa Cruz, CA, USA). The polyclonal anti-integrin β1 antibody was from Chemicon (Temecula, CA, USA). Alexa Fluor 488 Phalloidin and TO-PRO-3 were from Molecular Probes (Eugene, OH, USA). The horseradish-peroxidase (HRP)-conjugated sheep anti-mouse and sheep anti-rabbit antibodies were supplied from Amersham Biosciences (Buckinghamshire, UK). All other chemicals were reagent grade.

### 4.2. Patients and Tissue Immunofluorescence

RCC tissue samples were obtained from patients with a CT-confirmed renal mass and signed consent under an institutional review board-approved protocol (Decision n. 152/CE/2014 by the Ethical Committee at the University Hospital “Ospedali Riuniti” di Foggia). At the time of surgery, all the patients showed no evidence of disease (NED). Clinical and pathological findings were gathered for staging at operation. T stage was defined by pathological examination and the N and M components were defined according to pathological findings or by clinical data when applicable. Pretreatment clinical stage was established according to the 7th edition of the AJCC-UICC TNM classification [36]. The 2004 World Health Organization and Fuhrman classifications were used in order to attribute histological type and nuclear grade, respectively. The groups of selected patients at the primary diagnosis had the same histology of conventional (clear cell) RCC and were staged T1N0M0 (7 patients), T2N0M0 (1 patient), T3aN0M0 (3 patients), T3bN1M0 (1 patient). Median age was 61.6 years (range 45–78 years, F/M = 1:2). The median follow-up was 49.9 months (range 22–67). PatientsPatients’ characteristics are included in Table 2.

Removed tissues were immediately embedded in OCT compound Tissue-Tek (Sakura Finetek, CA, USA), snap-frozen and stored in liquid nitrogen until use. Portions of tissue samples were fixed in 10% buffered formalin and paraffin-embedded. Patients enrolled were divided into two groups: patients that developed metastases and patients with no evidence of disease at their last clinical evaluation.

The expression of NFATc2, NFATc3 and NFATc4 were evaluated by indirect immunofluorescence and confocal microscopy analysis on renal paraffin embedded serial sections, 4 μm-thickness, using specific antibodies against the different members of the NFAT family (Santa Cruz Biotechnologies, Santa Cruz, CA, USA).

The sections were incubated for 1 h in blocking-buffer (2% bovine serum albumin, 0.5% fetal bovin serum in PBS) and then with the primary antibodies (1:50 dilution) in a humidified container. The immune complexes were then identified incubating for 1 h at room temperature, the biopsies with the secondary antibodies Alexa Fluor 488 goat anti-mouse IgG-FITC conjugate (1:200 dilution), Alexa Fluor 488 rabbit anti-goat IgG-FITC conjugate (1:200 dilution), and Alexa Fluor 488 goat anti-rabbit IgG-FITC conjugate (1:200 dilution; Molecular Probes), to detect NFATc2, NFATc3 and NFATc4, respectively. The nuclei were stained with TO-PRO-3 (1:5000 dilution). The slides were then mounted in Gel/Mount (Biomeda, Milan, Italy) and sealed. Negative control was obtained incubating tissue-sections with the blocking-solution and then substituting the primary antibody with a non-immune poly-IgG serum. The specific-fluorescence was analyzed by confocal-laser-scanning-microscopy using the Leica TCS SP2 (Leica, Wetzlar, Germany) equipped with argon-krypton (488 nm) laser. Confocal images were taken at 500-nm intervals through the z-axis of the section, encompassing a total of 4 μm in depth. Images from individual optical planes and multiple serial optical sections were analyzed, and the images were sequentially scanned. Image analysis was performed on all acquired fields using the Image J software 1.33u (http://rsb.info.nih.gov/ij/ (accessed on 29 February 2008)).

### 4.3. Cell Isolation and Short Term Cultures of Human Tumors

RCC cell lines were isolated from kidney tissue samples of patients affected by RCC. Fresh tumor tissue obtained from the primary tumor was minced mechanically and subjected to enzymatic digestion with hyaluronidase 0.1%, collagenase IV 1% and Dnase I 0.02% for 20 min at room temperature. After passage through a 180 μm mesh, cells were cultured in RPMI 1640 supplemented with 2 mM L-glutamine, 100 IU/mL penicillin, 100 μg/mL streptomycin and 20% FBS. RCC cells were maintained in monolayer and passaged with 0.05% trypsin. Early passages were cryopreserved in FBS plus 10% DMSO.

### 4.4. Flow Cytometry and Apoptosis Assay

Anti-human CD40-FITC and β1-integrin-FITC conjugated antibody with their isotype control (BD Biosciences, San Jose, CA, USA) were used for immunofluorescent staining of RCC cell lines. Cells were washed and re-suspended in FACS buffer (phosphate- buffered saline pH 7.2, 0.2% bovine serum albumin, and 0.02% sodium azide) containing 3% human serum and incubated with fluorochrome-conjugated antibodies for 15 min at 4 °C, then washed with the same buffer before flow cytometric analysis.

Cell fluorescence was acquired using a Coulter EPICS XL flow cytometer and analyzed using WinMDI version 2.9 software.

For apoptotic analysis 1 × 10^6^ cells were double stained with 20 μL of 7-AAD Viability Dye and 10 μL of Annexin V-FITC for 15 min on ice in the dark (Annexin V-FITC/7-AAD Kit, Beckman Coulter, Fullerton, CA, USA) and then analyzed on a COULTER EPICS XL equipped with the SYSTEM II software. Annexin V-FITC and 7-AAD emissions were detected in the FL-1 (525 nm) and FL-4 (675 nm) channels, respectively, and their bi-parametric histogram has shown the presence of four distinct populations: the viable cells, the apoptotic cells, the secondary necrotic cells and necrotic cells.

### 4.5. CFDA-SE (5[6]-Carboxyfluorescein Diacetate Succinimidyl Ester) Cell Proliferation Assay

Cells were detached with 0.05% Trypsin-EDTA and labeled with 1 μM of CFDA-SE (CFSE, Molecular Probes Inc., Eugene, OR, USA) in PBS for 4 min at room temperature in the dark. CFSE loading was stopped with culture medium, cells were washed three times and seeded in six wells plates. CFSE was detected in FL1 with a logarithmic amplifier and analyzed on a COULTER EPICS XL equipped with the SYSTEM II software.

### 4.6. MTT Assay

The effect of CD40-ligation and cyclosporine A on RCC cell proliferation was analyzed with MTT assay, using the cell growth determination kit MTT based (Sigma). Briefly, cultured RCCs were digested with 0.05% trypsin and then were cultured in serum-free medium in 96-well culture plates (100 μL per well). RCCs were pre-incubated with cyclosporine A (5 μM) for 18 h and/or stimulated with sCD40L with the enhancer or the enhancer alone for 24 or 48 h and then supplemented with 10 μL MTT (5 mg/mL) and incubated for another 4 h. The supernatant was then discarded by aspiration and the RCC preparation was shaken with 100 μL MTT solvent for 10 min, before the OD value was measured at 570 nm.

### 4.7. Migration Assay

Migration of RCC cell lines upon sCD40L stimulation was determined using an in vitro model of wound repair [37]. In brief, confluent RCC monolayers were made quiescent in RPMI1640 for 24 h. The monolayers were then scraped with a razor blade, and cellular debris were removed by washing with RPMI1640. The cells were incubated in RPMI 1640 containing sCD40L (100 ng/mL) and sCD40L enhancer (1 μg/mL) or the enhancer alone for 24 h. In separate sets of experiments, cells were pre-incubated with cyclosporin (5 μM) for 18 h before adding sCD40L (100 ng/mL) and sCD40L enhancer (1 μg/mL) or the enhancer alone for 24 h. After the incubation the cells were fixed with 25% acetic acid/75% methanol and stained with hematoxylin. Cell migration was evaluated by counting the number of cells migrating into the denuded area with a phase-contrast microscopy under 63× magnification with a grid. Each condition was examined in duplicate with five representative fields for each plate being counted.

### 4.8. Western Blot Analysis

RCC cells were incubated 40 h in serum-free medium and then exposed to sCD40L (100 ng/mL) and sCD40L enhancer (1 μg/mL) or the enhancer alone for the indicated time periods. At the end of the treatment, the cell monolayer was lysed in RIPA buffer as previously reported [38]. Aliquots containing 40 μg of proteins from each lysate were subjected to SDS/PAGE on a 7.5% or 10% gel, according to the molecular weight of the protein of interest, under reducing conditions and then electro-transferred onto nitrocellulose membrane (Hybond^TM^ C, Amersham, Cologno Monzese, Italy). The membrane was blocked over-night at 4 °C with PBS plus 0.1% Tween-20 and 5% low-fat milk and then incubated with the relevant primary antibodies at the appropriate concentration. The membranes were then incubated for 1 h at RT with peroxidase-conjugated secondary antibodies. The membranes were washed three times at RT in TBS and then once with 0.1% SDS in PBS. The ECL enhanced chemiluminescence system (Amersham) was used for detection. The same membranes were then stripped and immunoblotted again with appropriate antibodies to normalize the protein quantity. The ECL enhanced chemiluminescence system (Amersham) was used for detection. Intensity of the bands was quantified using ImageJ software. Phosphorylated protein expression was normalized to total protein.

### 4.9. Transient Transfections and Luciferase Assay

Transient transfection was carried out by electroporation using the Gene Pulser II RF module (Biorad, Hercules, CA, USA) for the pLuc3XNFκB vector or by Lipofectamine (Invitrogen) for the pLuc3XAP1 vector, as previously described [38].

Briefly, 5 × 10^6^ RCC cells were re-suspended in half milliliter medium containing 7.5 μg of pLuc3XNFκB and 2.5 μg of pCMVβGal and kept on ice for 10min. Electroporation was carried out at 50 μF and 1.2 Kv. To determine the effect of sCD40L stimulation, the cells were rinsed once with RPMI1640 and triplicate wells were incubated with sCD40L (100 ng/mL) and sCD40L enhancer (1 μg/mL) or the enhancer alone in 1 mL of serum-free RPMI1640. Following incubation, the cells were lysed in 100 µL of reporter lysis buffer supplied with the Luciferase Reporter Assay System (Promega). The extracts were incubated at RT for 10 min and centrifuged at 12,000× *g* for 5 min. Twenty microliters of the supernatant were assayed for luciferase activity using a DIGENE DCR-1 luminometer (Abbot Laboratories, Abbot Park, IL, USA). Luciferase activity was normalized to β-galactosidase.

In separate sets of experiments 5 × 10^6^ cells were re-suspended in 500 μL of RPMI1640 without antibiotic for 24 h at 37 °C. 0.4 μg of pLuc3XAP1 were mixed with 1:1 diluted lipofectamine in Opti-MEM^®^ I Reduced Serum Medium (Invitrogen) for 30 min at RT and then added to the cells. After an incubation at 37 °C per 18 h, cells were scraped and lysed in 100 µL of reporter lysis buffer supplied with the Luciferase Reporter Assay System (Promega). The extracts were incubated at RT for 10 min and centrifuged at 12,000× *g* for 5 min. Twenty microliters of the supernatant were assayed for luciferase activity using a DIGENE DCR-1 luminometer (Abbot Laboratories).

### 4.10. Real Time PCR

NFATs, β1-integrin and ILK mRNA expression were investigated by real time PCR. Total RNA was isolated from RCC using TRIzol© reagent (Invitrogen, Carlsbad, CA, USA) and cDNA was synthesized by High Capacity cDNA Reverse Transcription Kit (Applied Biosystems, Foster City, CA, USA), according to the manufacturer’s instructions. The expression levels of the different genes were analyzed by real-time PCR on a 7500 Fast Real-Time PCR System (Applied Biosystems). Real Time PCR reactions were performed using Fast SYBR Green Master Mix (Applied Biosystems). Human ACTB (beta actin) gene was used as Endogenous Control (Applied Biosystems). Each sample was tested in triplicates and negative controls were also included. Quantitative values were obtained from the Ct (threshold cycle) data determined using default threshold settings. Gene expression data was normalized to human ACTB and relative quantification (RQ) was calculated with the 2^−ΔΔCt^ method. The data were presented as relative quantity (RQ) of target genes, normalized with respect to ACTB and a calibrator sample.

### 4.11. Confocal Analysis

RCC cells seeded onto coverslips were pre-incubated with curcumin (8 μM) for 18 h before adding sCD40L (100 ng/mL) and sCD40L enhancer (1 μg/mL) for the indicated time periods. Cells were then fixed in 4% paraformaldehyde for 15 min at 37 °C, and washed twice with PBS. Subsequently cells were incubated for 1 h at 37 °C with 3% BSA. Cells were then incubated with the monoclonal anti-c-jun antibody overnight at 4 °C. Cells were then washed three times with PBS and incubated with the secondary antibody Alexa Fluor 555 goat anti-mouse IgG-TRITC conjugate (1:400 dilution). In different sets of experiments, cells were incubated with sCD40L (100 ng/mL) and sCD40L enhancer (1 μg/mL) or sCD40L enhancer alone for the indicated time periods before adding the monoclonal anti-NFκB p65 antibody or the polyclonal anti-integrin β1 antibody overnight at 4 °C. Cells were then washed three times with PBS and incubated with Alexa Fluor 488 goat anti-mouse IgG-FITC conjugate (1:300 dilution) or Alexa Fluor 555 goat anti-rabbit IgG-TRITC conjugate (1:400 dilution), respectively, for 1 h at 4 °C in the dark. After washing, cells were incubated with phalloidin for 30 min at room temperature. Negative controls were performed by omitting the primary antibody and using non-immune mouse antiserum as first layer. The nuclei were stained with TO-PRO-3 (1:5000 dilution). Subsequently, cells were scanned using a Leica TCS NT confocal microscope with a 60X oil immersion objective. Cells were scanned midplane at 1024 × 1024 pixel resolution, and 4 serial identical sections were averaged.

### 4.12. Statistical Analyses

Data are presented as mean ± SD and compared by ANOVA. A *p* < 0.05 was considered significant.

## Figures and Tables

**Table 1:** Expression on RCC cell lines. RCC cell lines were isolated from kidney tissue samples of patients affected by RCC as described in methods. Data are expressed as percentage of expression.

Cell Line	Basal
**Name**	**% of Expression**
PRCC4	3.8
PRCC5	0.77
PRCC6	2.2
PRCC7	48.56
PRCC11	64.47
PRCC18	99.78
PRCC20	19.15
PRCC21	99.02
RCC1851	49.03
Mean ± SD	42.97 ± 39.43

**Table 2:** Patients’ characteristics. TKI: Tyrosine-kinase inhibitor; RT: radioteraphy; S: Surgery; Mets: metastatic disease; NED: no evidence of disease.

Patient	Age/Sex	Stage	Grade	Adjuvant Treatment	Last Evaluation	Follow-Up (Months)
1	63 M	T1N0M0	2	/	NED	55
2	55 M	T1N0M0	1	/	NED	67
3	74 M	T1bN0M0	2	/	NED	65
4	46 F	T1aN0M0	2	/	NED	52
5	76 F	T1N0M0	3	/	NED	39
6	53 F	T1bN0M0	3	/	NED	53
7	66 M	T1N0M0	3	/	Mets, liver-chest	32
8	45 M	T2N0M0	2	TKI, RT, S	Mets, bone	22
9	78 F	T3bN1M0	3	/	Mets, lung-spleen	62
10	53 M	T3aN0M0	3	S, TKI	Mets, lung-spleen	57
11	69 M	T3aN0M0	3	S, TKI	Mets, lung-spleen	41
12	62 M	T3aN0M0	1	RT, S	Mets, bone	54

## Data Availability

All data generated during this study is included in this published article.
